# Spatiotemporal drivers of water quality and phytoplankton communities in a cyanobacteria-dominated reservoir provide management insights

**DOI:** 10.1007/s10661-025-14258-1

**Published:** 2025-06-24

**Authors:** Linnea A. Rock, William W. Fetzer, Lindsay S. Patterson, Samuel J. Sillen, Ron Steg, Annika W. Walters, Sarah M. Collins

**Affiliations:** 1 Department of Zoology and Physiology, University of Wyoming, Laramie, WY USA; 2 Program in Ecology and Evolution, University of Wyoming, Laramie, WY USA; 3 Water Quality Division, Wyoming Department of Environmental Quality, Cheyenne, WY USA; 4 Department of Geology and Environmental Science, University of Pittsburgh, Pittsburgh, PA USA; 5 U.S. Geological Survey Wyoming Cooperative Fish and Wildlife Research Unit, University of Wyoming, Laramie, WY USA

**Keywords:** Cyanobacteria, Reservoir, Monitoring, Spatiotemporal, Nutrients, Phytoplankton

## Abstract

Reservoirs serve critical roles providing drinking water, irrigation, flood control, hydropower, recreation, fisheries, and aquatic habitat. Yet their physical position, complex shape, and large watersheds make reservoirs especially susceptible to eutrophication and harmful algal bloom (HAB) production. Boysen Reservoir, WY, is a high priority for proactive nutrient management because it is an important source for drinking water and recreation, and has a history of toxic cyanobacterial blooms. We combined four years of comprehensive monitoring efforts by state and federal agencies to characterize the spatiotemporal patterns of nutrient inflow, internal water quality dynamics, and phytoplankton community shifts in Boysen Reservoir. We found nutrient inflow was hydrologically driven, with snowmelt runoff transporting high nutrient loads. Our findings suggest physicochemical and nutrient conditions of the reservoir were strongly different between the furthest reaches of the reservoir, but less variable among the intermediate sites. Space did not play a role in phytoplankton community dynamics, but time was an important factor. Cyanobacteria dominated phytoplankton communities by mid-summer across the reservoir and were driven mainly by temporal physicochemical conditions, like stratification and water temperature. The two most dominant phytoplankton taxa across the four years of sampling were N-fixing, toxin producing cyanobacteria. Extensive monitoring efforts and data analyses can illuminate strategies to safeguard water resources via understanding the drivers of water quality changes and HAB production.

**Supplementary Information:**

The online version contains supplementary material available at 10.1007/s10661-025-14258-1.

## Introduction

Eutrophication—or dense primary productivity usually associated with excessive nutrients—of freshwaters can be detrimental to aquatic ecosystem health. Consequences include decreased levels of dissolved oxygen, formation of toxic compounds, changes in abundance and composition of various aquatic organisms, and loss of biodiversity (Camargo & Alonso, 2006; Reid et al., 2019). Eutrophication also causes serious economic damages across the US every year, conservatively estimated at $2.2$$-$$4.6 billion (Hudnell, 2010). Costs are based on water treatment needs, loss of recreation, fisheries, biodiversity (Dodds et al., 2008), and property values (Mamun et al., 2023). Nutrients exceed reference levels in freshwaters across the US (Dodds et al., 2008), and eutrophication remains one of the greatest threats to freshwaters globally (Smith & Schindler, 2009). Eutrophication has been linked to harmful algal blooms (HABs) and poor water quality (Reid et al., 2019; Camargo & Alonso, 2006). Globally, increased awareness and monitoring have given the impression of intensifying eutrophication (Hallegraeff et al., 2021). There is conflicting evidence and rationale for intensification of algal blooms in lakes globally. A study examining bloom data in large lakes globally found most of the study lakes had an increase in peak summertime bloom intensity since the 1980s (Ho et al., 2019). Surprisingly, other broad-scale studies including hundreds to thousands of lakes suggest that lakes do not show signs of algal bloom intensification (Wilkinson et al., 2022) or significant degradation in water quality (Oliver et al., 2017); rather, water clarity has increased in US lakes since the 1980s (Topp et al., 2021). Regardless, there is a pressing need to evaluate whether current monitoring programs are effectively capturing water quality trends in vulnerable freshwaters.

HABs result from an overgrowth of phytoplankton, some of which can produce toxic compounds (cya-notoxins) that threaten human and animal health (Carmichael, 2001). The risk is especially acute when cyanotoxins are present in drinking water supplies (Funari & Testai, 2008). Even when toxins are absent, blooms can degrade water quality, diminishing recreational and economic value of freshwaters (Angradi et al., 2018). HABs often arise in areas with high nutrient loading due to anthropogenic activities, like agricultural runoff or urbanization, which increase nitrogen and phosphorus availability. Climate change exacerbates this issue by altering the hydrological cycle and raising water temperatures, both of which promote nutrient accumulation in freshwater systems (Rastogi et al., 2015). For instance, in the upper midwest, extreme precipitation events are strongly linked to phosphorus loading, which directly fuels the growth of HABs (Carpenter et al., 2022). Physical dynamics, such as lake stratification and mixing, combined with the ability of phytoplankton to adapt to changing light conditions, also influence photosynthesis and bloom formation (Tilzer & Goldman, 1978). Changes in ice cover and stratification due to warming may further alter bloom timing and magnitude by affecting phytoplankton productivity and community composition (Winder & Sommer, 2012). As a result, HABs not only pose significant ecological threats but also carry serious economic and public health consequences.

Eutrophication has received less attention in western US reservoirs compared to natural lakes in eastern regions of the US, yet the effects on these waterbodies are equally important. The actual incidence of eutrophication and HABs may be difficult to document especially when data are biased (Stanley et al., 2019) and incomplete in sparsely populated regions such as the western US. Satellite imagery in the western US suggests there is no evidence of declining water quality and that most lakes in this region have been static in clarity since the 1980s. However, nearly a third of lakes and reservoirs in this region are eutrophic (Oleksy et al., 2022; Sillen et al., 2024). Reservoirs are especially prevalent in the western US because of the semi-arid climate and high water demand, and they have played a large role in the development of this region as they provide for critical needs such as agricultural irrigation, flood control, and drinking water, along with being important for recreation and aquatic habitat (Marzolf & Robertson, 2006).

Reservoirs are particularly susceptible to eutrophication because they are often more intimately connected to the landscape than natural lakes. Specifically, reservoirs drain larger catchments than natural lakes, have larger, more complex perimeters, and sit low in the landscape, increasing their potential to integrate nutrient pollution as settling basins (Hayes et al., 2017). Because of their complex physical structure, reservoirs can be divided into three functional zones illustrating key areas that vary in their physical structure and biogeochemical function: riverine, transitional, and lacustrine. Riverine zones are typically more influenced by the main tributary, with higher turbidity, nutrients, and productivity. Lacustrine zones function more like natural lakes and are likely to be relatively less productive, nutrient-rich, and turbid than riverine zones. Transitional zones are intermediate between the riverine and lacustrine zones (Kennedy et al., 1985). Consideration of the three zones is critical for monitoring water quality as they may demonstrate distinct responses to anthropogenic, climate, or other influences. Further, reservoirs are unique in their heterogeneity and setting; thus, local-scale studies are important to understand the drivers of eutrophication.

In this study, we investigated nutrient and phytoplankton dynamics in Boysen Reservoir, a reservoir in west central Wyoming, USA, that serves the needs of the surrounding communities with drinking and irrigation water, fisheries, recreation, and as aquatic habitat. Boysen Reservoir has been consistently listed by the Wyoming Department of Environmental Quality (DEQ) and Wyoming Department of Health for advisories due to HABs and cyanotoxin presence since monitoring began in 2017. To better understand how monitoring can provide insights into the water quality dynamics driving algal blooms in this reservoir, we investigated spatiotemporal heterogeneity of nutrient inflow, water quality, and phytoplankton communities using data collected by the DEQ alongside US Department of the Interior, US Geological Survey (USGS), and Bureau of Reclamation (BoR) stream monitoring. We specifically aimed to answer the following questions: (1) To what extent do spatiotemporal patterns in reservoir water quality dynamics contribute to the proliferation of cyanobacteria? (2) What are the key drivers of phytoplankton community shifts, and how do these factors influence cyanobacterial dominance in the reservoir? We hypothesized that both water quality dynamics and phytoplankton communities would differ across reservoir zones and as the growing season (May–October) progressed. Our approach, which assessed heterogeneity in both water quality and phytoplankton dynamics, illustrates how comprehensive spatiotemporal monitoring and data evaluation can lead to efficient monitoring programs that move beyond detection of HABs toward focusing on drivers of water quality that can inform action to safeguard our freshwaters.

## Materials and methods

### Study location and data collection

Boysen Reservoir (Fig. 1) is located on the Wind River, 23 km south of The Wedding of the Waters, where the Wind River changes names to Bighorn River. The Reservoir is at elevation 1463 m, with a surface area of 7920 ha and has a maximum depth of 39.6 m. Boysen Reservoir drains a large (1,994,291 ha), complex watershed including headwaters originating in the Wind River Range and Absaroka Range of the Rocky Mountains. In 2020–2023, DEQ collected monthly data May–October from seven locations along the reservoir (WYDEQ, 2023). For each reservoir zone, there was a primary pelagic sampling location (Riverine Pelagic, Freemont 1; Transitional Pelagic, Sand Mesa; and Lacustrine Pelagic, Dam), with the remaining sampling sites acting as gradients between the zones. Surface grab collections included Secchi depth, pH, dissolved oxygen (DO), specific conductivity (SpC), chlorophyll-a, total phosphorus (TP), total nitrogen (TN), orthophosphate (PO_4_^3-^ as P), total ammonium (NH_4_^+^ as N), and nitrate (NO_3_^-^ as N). DEQ additionally collected water column profile data for SpC, DO, pH, and water temperature. We refer to the collective physicochemical parameters as “water quality.” Phytoplankton were collected at one-half the Secchi depth at each sampling event. Phytoplankton communities were identified to genus using a 300 natural counting unit target. Shoreline samples were collected as part of Wyoming’s Recreational HAB Advisory Program and tested for toxins, including anatoxin-a, cylindrospermopsin, total microcystins, and saxitoxin.

**Fig. 2:**
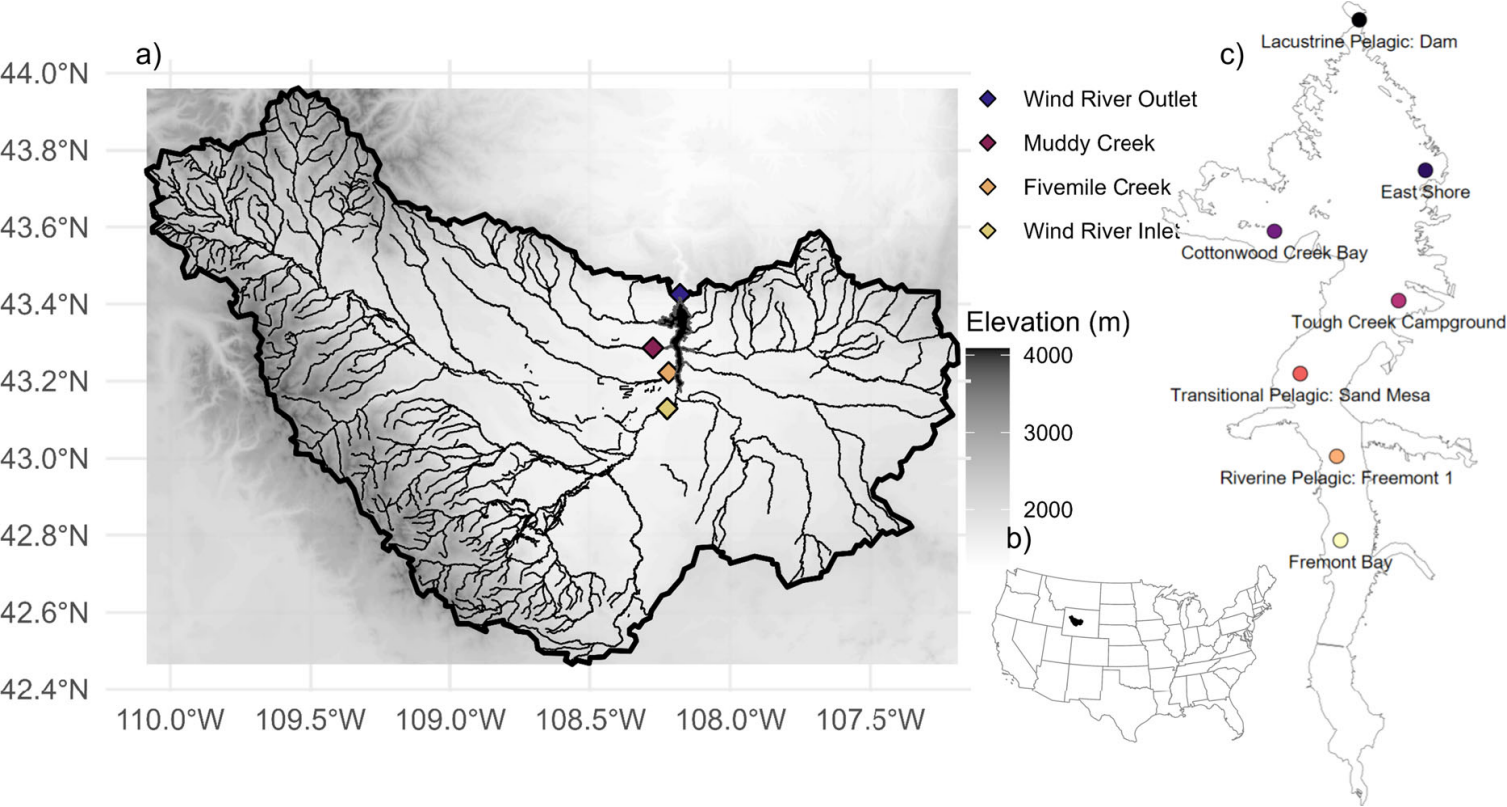
Map displaying the location of the sampling locations in and around Boysen Reservoir. **a** Watershed map of Boysen Reservoir showing inflows with mountainous, high elevation areas indicated by lighter background color. The inflows and outflow USGS and BoR river data sampling sites indicated via colored, diamond points. **b** The US map with location of Boysen watershed depicted in black. **c** DEQ sampling sites across Boysen Reservoir. Direction of flow is toward the north

The USGS monitored the three major tributaries (Wind River Inlet (USGS, 2024d), Muddy Creek (USGS, 2024b), and Fivemile Creek (USGS, 2024a)) continuously for discharge and monthly for nutrients (TN, TP, ammonium as N, nitrate as N, and phosphate as P). The USGS also monitored the outlet (Wind River Outlet (USGS, 2024c)) monthly for nutrient concentrations. Wind River Outlet continuous discharge and Boysen Reservoir storage were monitored by the BoR (BoR, 2024a, b). USGS data were downloaded from the National Water Information System via the dataRetrieval R package (De Cicco et al., 2018); Bureau of Reclamation data were downloaded via the Reclamation Information Sharing Environment database; and DEQ data were obtained via open records requests.

### Data processing and analyses

All data analyses were performed in the R programming language version 4.4.2 (R Core Team, 2024). The tidyverse (Wickham et al., 2019), lubridate (Grolemund & Wickham, 2011), and patchwork (Pedersen, 2024) packages were used for a majority of data analyses and visualizations. The terra (Hijmans, 2024), tidyterra (Hernangómez, 2023), sp (Bivand et al., 2013), sf (Pebesma, 2018; Pebesma & Bivand, 2023), raster (Hijmans, 2023), ggspatial (Dunnington, 2023), and usmap (Lorenzo, 2024) packages were used for creating map visualizations and working with bathymetry data.

Monthly tributary and outlet nutrient loads (kg month^-1^) were calculated by multiplying monthly nutrient concentrations (mg L^-1^) by the average monthly discharge (L sec^-1^) and a monthly time interval (2.682 * 10^6^ sec month^-1^). Muddy Creek and Wind River Inlet had a lapse in continuous discharge data from January–September 2020. We used the available historical data to estimate a monthly average to fill in the missing data. We then estimated nutrient retention or within-reservoir use as the percent of tributary loading that exited the reservoir via the outlet. When retention was <0, it indicates there was a greater nutrient export out of the reservoir than what entered via the tributaries. There were many gaps in the nutrient content data that varied among the tributaries and are reflected as blank spaces for nutrient loading.

Due to the frequency of missing nutrient data in the tributaries, we pursued the potential to use discharge as a proxy for nutrient loading in our analyses. Monthly nutrient loading of TN and TP was regressed against total discharge—as the sum of average monthly discharge from the three tributaries. We found strong correlations between nutrient loading (*R*^2^=0.98 for TN and *R*^2^=0.81 for TP, Figure S1).

All reservoir concentration data that were reported as below the laboratory detection limit were replaced with half the detection limit for use in the analyses. Replicates were averaged before analyses occurred. Stoichiometric ratios of N:P were calculated as molar ratios. Less than 0.5% of raw surface water quality data were outliers caused by measurement errors and were outside of ecologically realistic ranges. To replace outliers, we averaged surface measurements during that month and at that location from the other years to estimate. For both in-reservoir and river samples, we summed nitrate and ammonium concentrations and refer to it as inorganic nitrogen (IN) throughout the manuscript. Hypolimnion SpC, DO, pH, and temperature data used in the manuscript correspond to the deepest measurements at each sampling location and date.

Schmidt stability, a measure of resistance to mixing (Idso, 1973; Schmidt, 1928), was calculated for each sampling location and date using the Schmidt stability function from the rLakeAnalyzer package (Winslow et al., 2019). The rLakeAnalyzer package uses water temperature depth profiles, hypsometry (cross sectional areas and corresponding depths), and salinity to calculate stability (Eq. 1, Idso (1973)).

1 $$\begin{aligned} S_T=\frac{\textrm{g}}{A_S} \int _0^{z_D}\left( z-z_v\right) \rho _z A_z \partial _z \end{aligned}$$

where,

$$\begin{aligned} S_T=Schmidt\, stability \nonumber \\ g=Acceleration\, due\, to\, gravity (constant) \nonumber \\ A_s=Surface\, area\, of\, the\, lake (constant) \nonumber \\ z_D=Maximum\, depth\, of\, location \nonumber \\ z=Depth\, at\, any\, given\, interval \nonumber \\ z_v=Depth\, to\, center\, volume\, of\, location \nonumber \\ \rho _z=Density\, of\, water\, at\, depth\, z \nonumber \\ A_z=Area\, at\, depth\, z \nonumber \end{aligned}$$

Hypsometry was developed using bathymetry data of the reservoir by rounding the continuous depth data to 0.5 m from the surface to the deepest point (24.5 m), and the continuous areas were summed at those aggregated depths. *z*_v_ is the center volume at each location, $\rho$_z_ is calculated at each location’s depths using water temperature associated with that depth and the salinity, and *A*_z_ corresponds to the cross sectional areas at each depth. Salinity was set to 0 for all locations and dates based on specific conductivity values suggesting salinity values would be negligible.

The phytoplankton sampled across Boysen Reservoir were categorized as either cyanobacteria or other and then calculated the percent of cyanobacteria density (cells L^–1^) for each month and sampling location. We evaluated the relative abundance ($\alpha$ diversity as %) of phytoplankton varieties at each location and sampling date as well as the species diversity (H) as Shannon-Wiener index (Eq. 2). H is a metric that describes overall species richness and relative abundance (Hauer & Lamberti, 2017). Cyanotoxin sampling occurred along the shoreline when a harmful algal bloom was suspected. We presented these data at the nearest of the seven sampling locations in this manuscript, which we determined using the gdistance package (Etten, 2017). Shortest locations were determined as the path distance traveled via water. Distances ranged from 0.6–3.7 km, with an average distance of 1.3 km.

2 $$\begin{aligned} H^{\prime }=-\sum _{i=1}^s p_i \ln p_i \end{aligned}$$

where,

$$\begin{aligned} H^{\prime }=Species\, diversity\, index \nonumber \\ s=Number\, of\, species \nonumber \\ p_i=Proporation\, of\, individuals\, of\, species\, i \nonumber \end{aligned}$$

#### Exploring spatiotemporal water quality patterns

We employed principal component analysis (PCA) to explore multivariate similarities and differences in water quality parameters across the seven sampling locations. We further used principle components (PCs) to determine which axes were best to visualize spatial patterns. We included TP, phosphate, TN, IN, TN:TP, IN:phosphate, surface and hypolimnion SpC, surface and hypolimnion DO, surface and hypolimnion pH, surface and hypolimnion temperature, Secchi depth, Schmidt stability, maximum depth, and Shannon-Wiener index of phytoplankton communities (*H*; Eq. 2) in the PCA. All data were scaled prior to developing the correlation matrix for the PCA. We then examined the loadings and variance explained to choose which principle components (PC) to further explore across space and time. We performed step-wise forward model selection using BIC from the MASS package (Venables & Ripley, 2002) to compare how our best PCs correlated with latitude. Latitude was an appropriate proxy for space as the reservoir runs south to north, with the seven locations spaced along to capture the riverine, transitional, and lacustrine zones. This approach allowed us to reduce the water quality data to a more limited number of variables (PCs) for which variation was correlated with latitude for the most effective visualization of spatial patterns. We visualized the relationship between PCs for all site locations over time to characterize how water quality changes over the growing season.

**Fig. 3:**
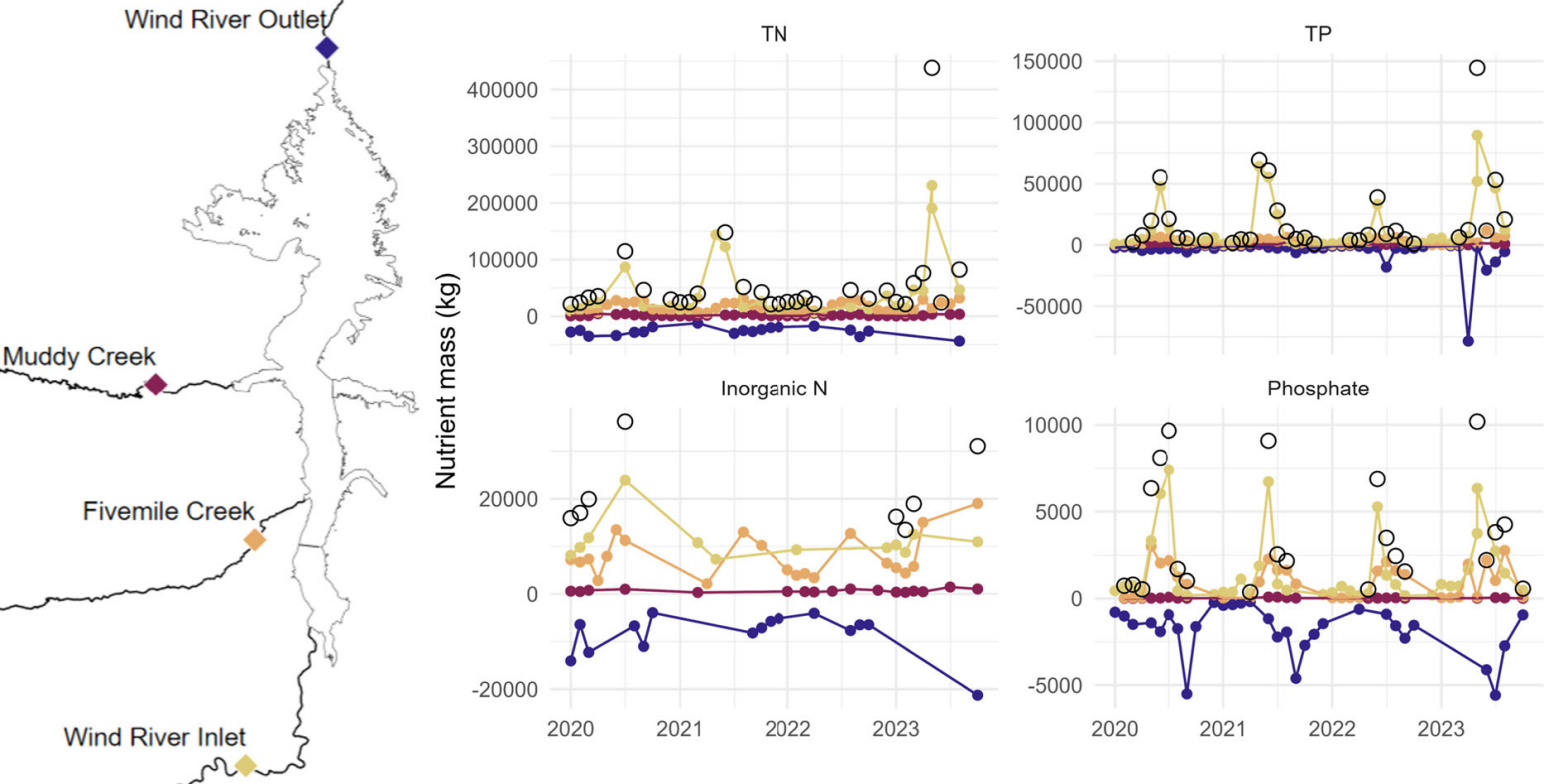
Total nitrogen (TN), total phosphorus (TP), inorganic nitrogen, and phosphate monthly mass loading by tributary and export via the outlet. Open circles depict the sum of monthly loading where we had complete tributary loading data. Legend shows locations of each tributary and the outlet

#### Assessing variability in phytoplankton communities

We investigated heterogeneity ($\beta$ diversity) of phytoplankton communities using nonmetric multidimensional scaling (NMDS) of Bray-Curtis dissimilarities of each sampling location and date using the metaMDS function from the vegan package (Oksanen et al., 2024). We then used the envfit function from the vegan package with 999 permutations to explore both the intrinsic (phytoplankton varieties) and extrinsic (water quality parameters) factors that co-vary with the phytoplankton community distribution pattern. Further, we used the adonis2 function from vegan with 999 permutations to individually test each extrinsic variable for significance in explaining variation in the Bray-Curtis dissimilarities of the phytoplankton communities (PERMANOVA). Extrinsic variables used in the models were date, year, month, latitude, longitude, average monthly reservoir storage, phosphate, IN, TP, TN, TN:TP, IN:phosphate, surface SpC, surface DO, surface pH, surface temperature, Secchi depth, Schmidt Stability, maximum depth, Shannon-Wiener index, and total discharge (as a proxy for nutrient loading).

## Results

### Nutrient loading and retention

Nutrient loading into Boysen Reservoir was highest in the furthest upstream tributary (the Wind River Inlet), followed by the next upstream tributary (Fivemile Creek), and lowest in Muddy Creek (Fig. 2). The high loading was driven by streamflow, which was greatest in Wind River Inlet (Figure S2); however, nutrient concentrations tended to be higher in the smaller tributaries (Figure S3). There was typically greater nutrient inflow than outflow, with the highest inflows occurring early in the growing season, often June or earlier during peak streamflow due to snowmelt (Figs. 2 and S1). We observed high variation among monthly nutrient retention within and across nutrients and forms. Monthly TN retention in the reservoir can be as high as 95% and as low as $-$132%, with a median of 53%. TP retention varied from 96 to $-$643%, with a median of 15%. Monthly IN retention had a median of 65% and varied from 38 to 88%. IN was never exported more than retained within Boysen Reservoir. Phosphate varied between $-$544 and 90%, with a median retention of 11%. TN retention has a strong linear relationship with time, in which retention increased through the year (slope = 17.52% month^-1^, *p* < 0.001, *R*^2^ = 0.63). TP, IN, and phosphate follow non-linear patterns over time (Figure S4). TP retention, while there was no significant linear trend (slope = $-$8.92% month^-1^, *p* = 0.4), did generally follow the opposite pattern as TN retention. Furthermore, the sum of percent retention annually, i.e., what percentage of nutrients retained within the reservoir that year, were opposite for TN and TP each year. We mainly observed TN retention and TP export. In 2020, annual retention of TN was $-$284% while TP was 148%; in 2021, TN retention was 318% and TP retention was $-$206%; in 2022, TN retention was 216% and TP retention was $-$295%; and in 2023, TN retention was 53% and TP retention was $-$753%.

Nutrient concentrations within the reservoir tended to be highest in the riverine zone (Fremont Bay - Riverine Pelagic: Freemont 1) of Boysen Reservoir, though differences were marginal across all seven sampling sites (Fig. 3). TN concentrations increased over the sampling season, following the retention pattern. TP showed high concentrations accompanying high tributary loading early season and was lowest mid-season, followed by accumulation later in the season. IN was often at or below detection level. Phosphate trends matched TP trends.

**Fig. 4:**
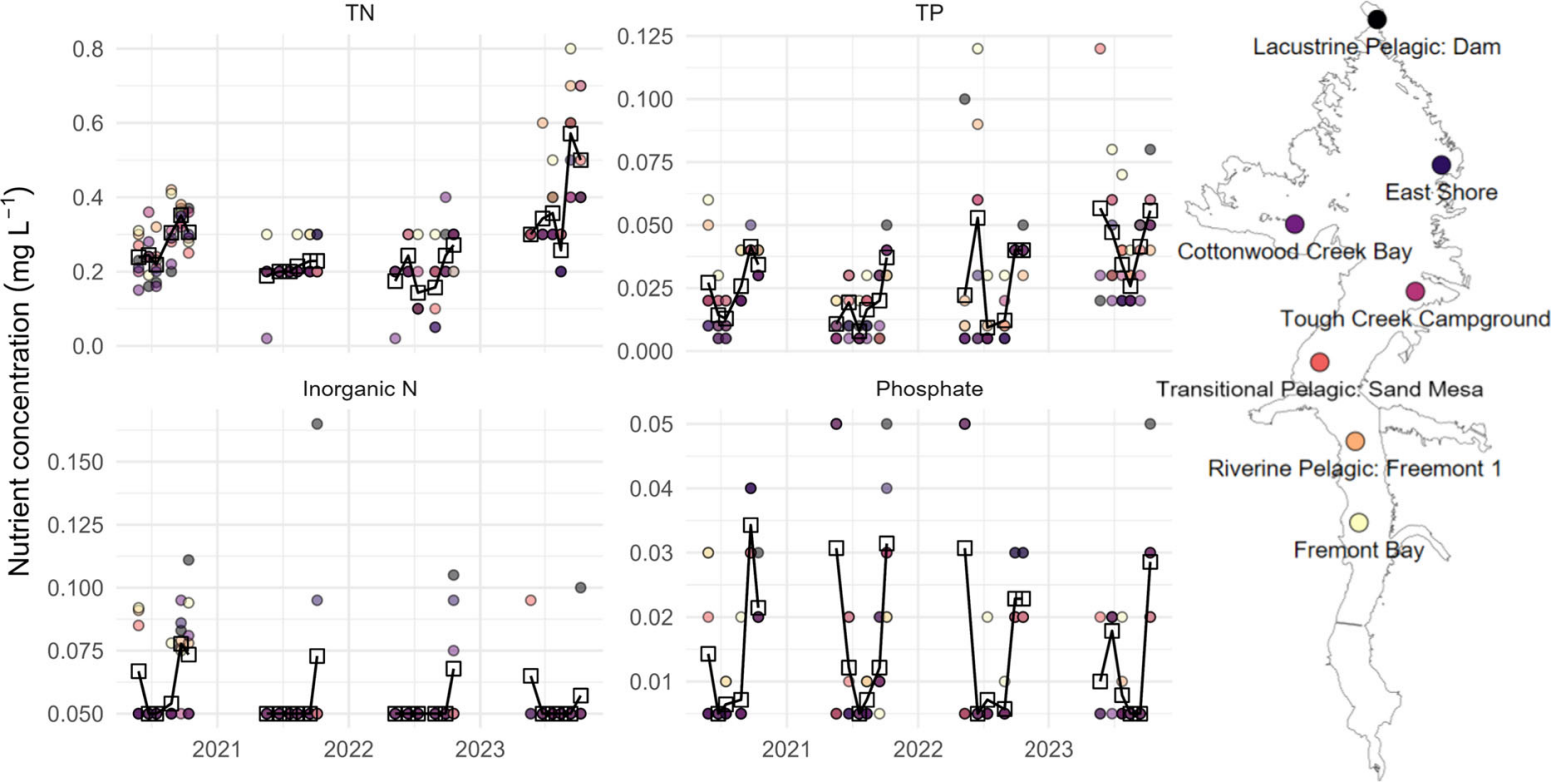
Total nitrogen (TN), total phosphorus (TP), inorganic nitrogen, and phosphate monthly concentrations distinguished by reservoir sampling location. Open squares depict the monthly average concentration in the reservoir. The sampling site legend is plotted over the outline of Boysen Reservoir for context on the position of each site

**Fig. 5:**
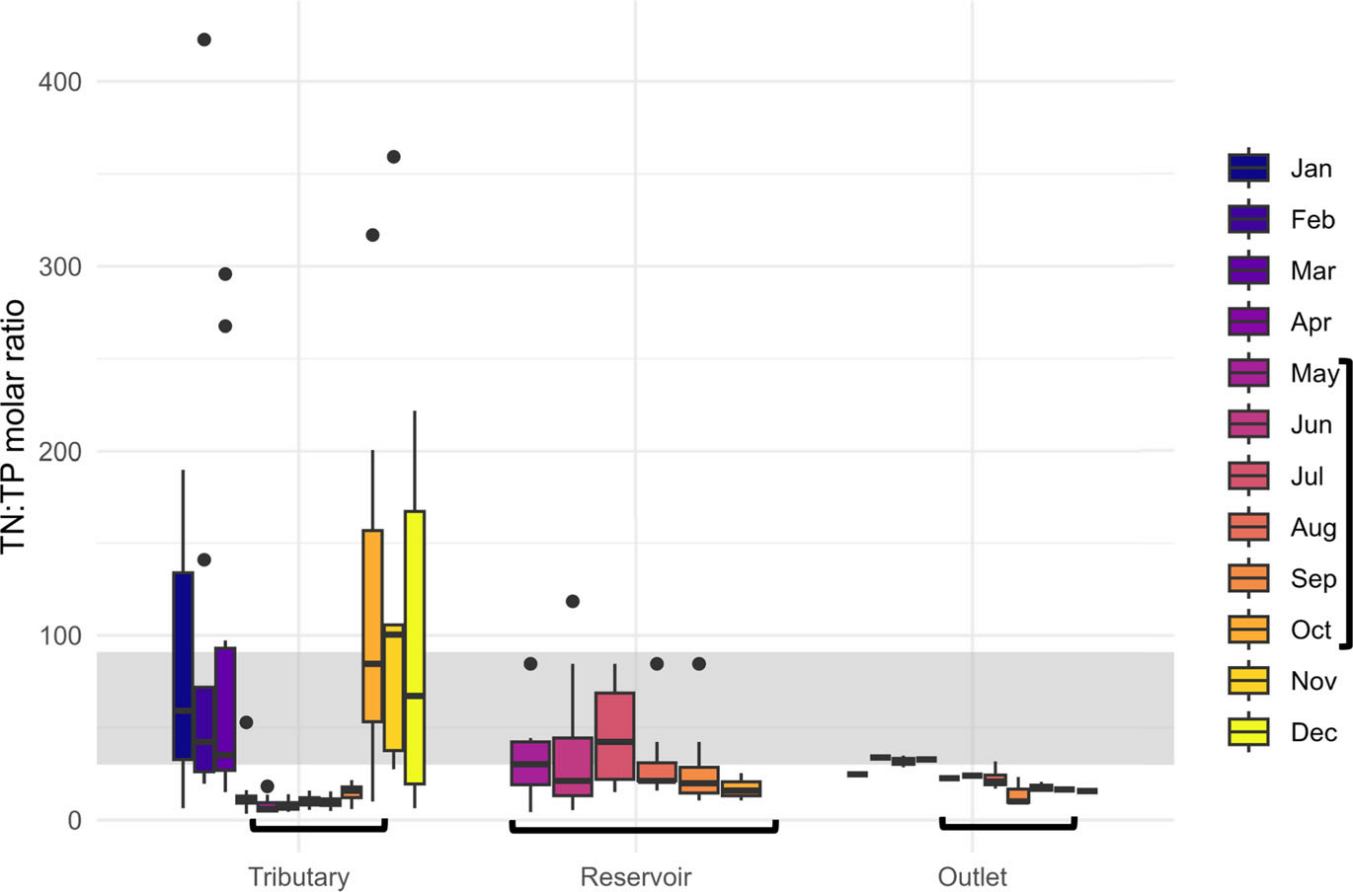
Monthly total nitrogen:total phosphorus molar ratios aggregated by all tributaries, reservoir locations, and the outlet. Black brackets at the bottom of plot indicate months of the year when Boysen Reservoir was monitored for water quality by the WY DEQ (May-October). The horizontal shaded area is the range of N:P values at which primary production may be co-limited by N and P or other factors (TN:TP 30-91, see Figure S5). Above the shaded range, we expect P-limited productivity, whereas below it, we expect N-limitation

Primary production in Boysen Reservoir was most often N-limited, or co-nutrient (N and P) limited in accordance with several N:P ratios established in the literature (Figs. 4, S5) (Bergström, 2010; Downing & McCauley, 1992; Forsberg, 1980; Ptacnik et al., 2010; Redfield, 1958; Rhee & Gotham, 1980; Sakamoto, 1966; Smith, 1982). During May–September, in-reservoir and outlet N:P ratios are higher than the tributary ratios. Reservoir and outlet ratios were similar, but with a higher range in ratios over the reservoir locations than the single outlet. In October, tributary N:P ratios were greater than the in-reservoir or outlet ratios. Outlet ratios remained consistent throughout the full year, likely due to the moderating effect of the reservoir. We observed much greater N:P ratios in the tributaries October–March than April–September.

### Spatiotemporal water quality dynamics

PCA and step-wise multiple linear modeling revealed PCs 1 and 3 were the best to visualize spatial variance (PC1 + PC3 explained 35% variance in PC space and 43% of variance across reservoir latitude: latitude PC1+PC3, $p<0.0001$). PC1 represents variability of phosphate, hypolimnion DO, IN: phosphate, surface water temperature, and Schmidt stability, while PC3 represents variability of Secchi depth, Schmidt stability, maximum depth at sampling, and hypolimnion water temperature (Fig. 5). We present the same PC axes with the primary loadings described within each month of the growing season. In May, there was less variability among the parameters described by PC3 than PC1, with greater phosphate and hypolimnion DO in the lower sections of the reservoir. In June, sites began shifting left on PC1, depleting phosphate and hypolimnion DO while increasing IN:phosphate, surface temperature, and Schmidt stability. In July and August, there was a negative relationship between the two PCs, such that higher phosphate and hypolimnion DO were associated with shallower sites with lower Secchi depth, Schmidt stability, and higher hypolimnion temperatures but lower surface temperatures. The variation in PC1 and PC3 corresponded to the spatial location of sites with Lacustrine Pelagic Dam having lower loadings on PC1 and higher loadings on PC3, while Fremont Bay experienced the inverse. In September and October, the pattern is more static in PC1, but loadings on PC3 increased with latitude, with Lacustrine Pelagic Dam having the highest loadings. Overall, the Lacustrine Pelagic Dam (lacustrine zone) and Fremont Bay (riverine zone) showed distinction from each other, and there may be less variability between the intermediate zone sites and the shore/bay sites than when comparing the three zones overall.

**Fig. 6:**
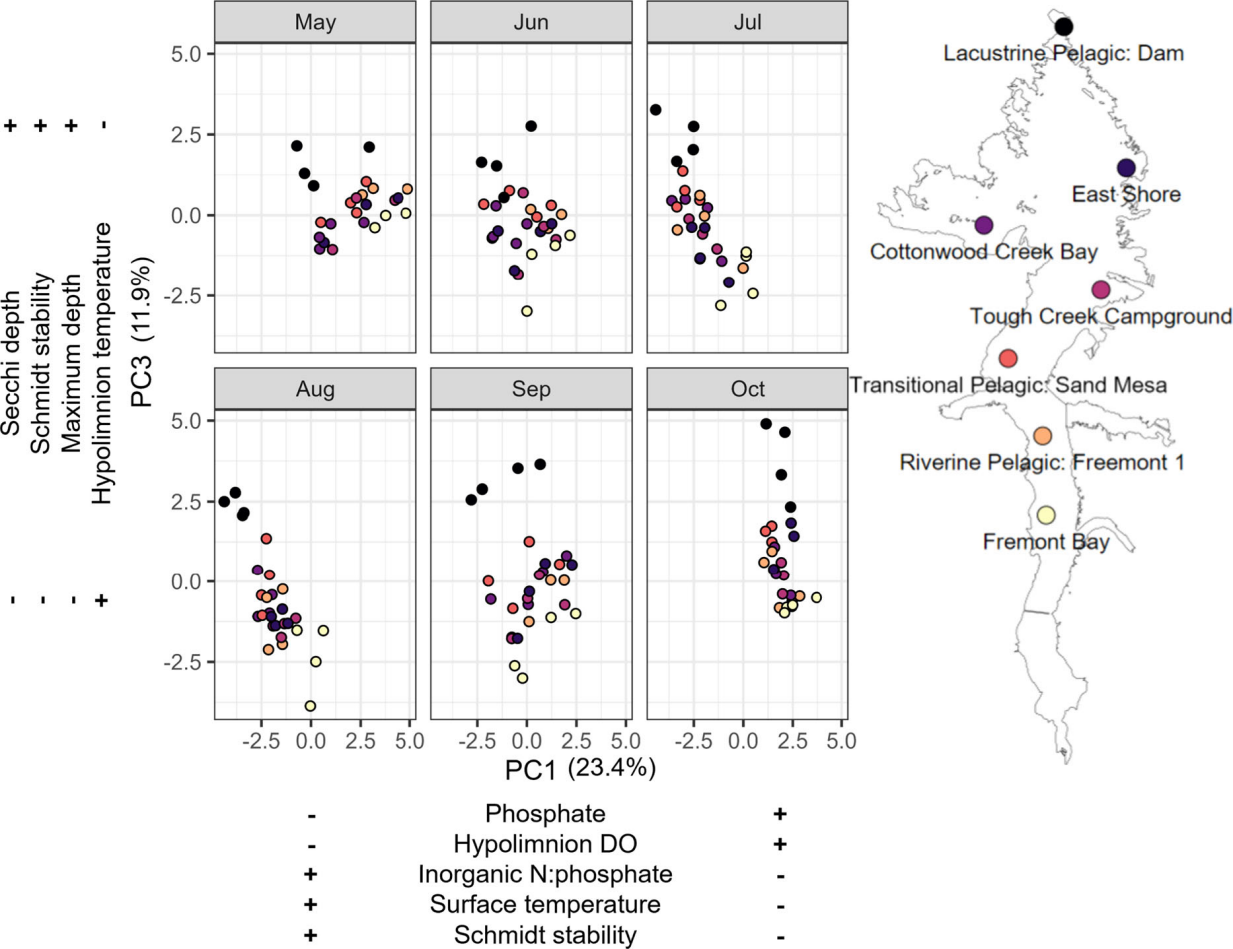
Principle component 3 vs principle component 1 with the primary loadings and their directions listed below PC1 on the *x*-axis and to the side of PC3 on the *y*-axis. The panels show PCs plotted monthly with each sampling site represented by a distinct color. The loadings correspond to the PCs, not to the faceted months. Each month displays the same PC axes. The sampling site legend is plotted over the outline of Boysen Reservoir for context on the position of each site

### Phyotplankton community dynamics

**Fig. 7:**
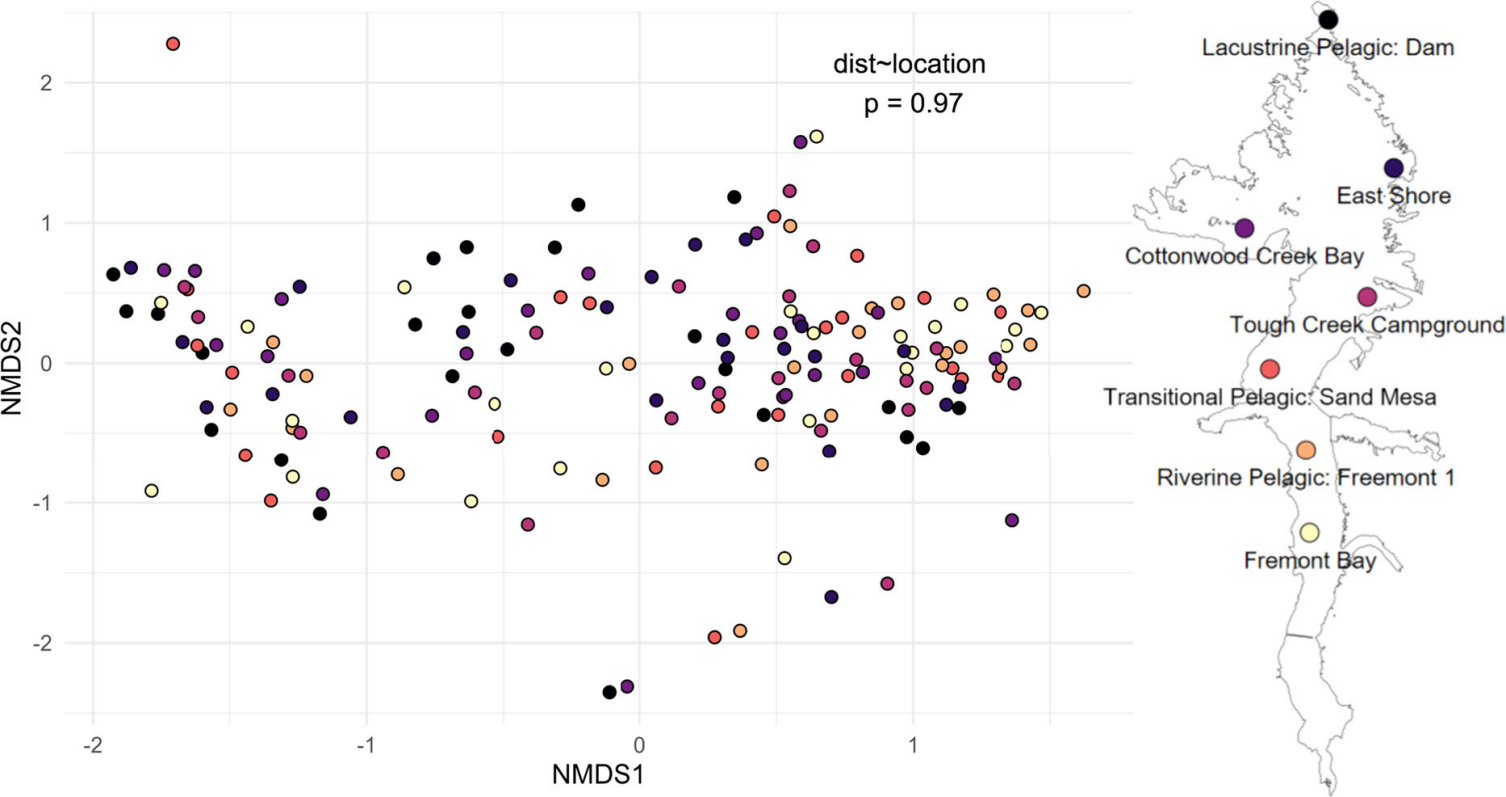
Beta diversity dispersion of phytoplankton communities in Boysen reservoir, with each point representing a location and date. Diversity points colored by sampling location, displaying the lack of spatial importance on diversity dynamics (PERMANOVA, dist location, *p*=0.97). The sampling site legend is plotted over the outline of Boysen Reservoir for context on the position of each site

Surprisingly, phytoplankton community diversity had no coherent spatial variability, and the communities did not differ across zones of the reservoir (PERMANOVA dist site, *p*=0.97, Fig. 6). However, phytoplankton community diversity was strongly driven by both time and the cyanobacteria percent of total community biomass, both of which were consistent across years (PERMANOVA, *p*=0.021 and *p*=0.037, respectively, Fig. 7). There were 24 phytoplankton varieties that significantly explained the beta diversity below alpha=0.05. We present the top eight—significant below alpha=0.001—in Fig. 7a, half of which are cyanobacteria, followed by three diatoms, and one golden algae. Of particular note, *Aphanothece* tended to dominate across the reservoir mid-season and was succeeded by *Aphanizomenon* late season.

**Fig. 8:**
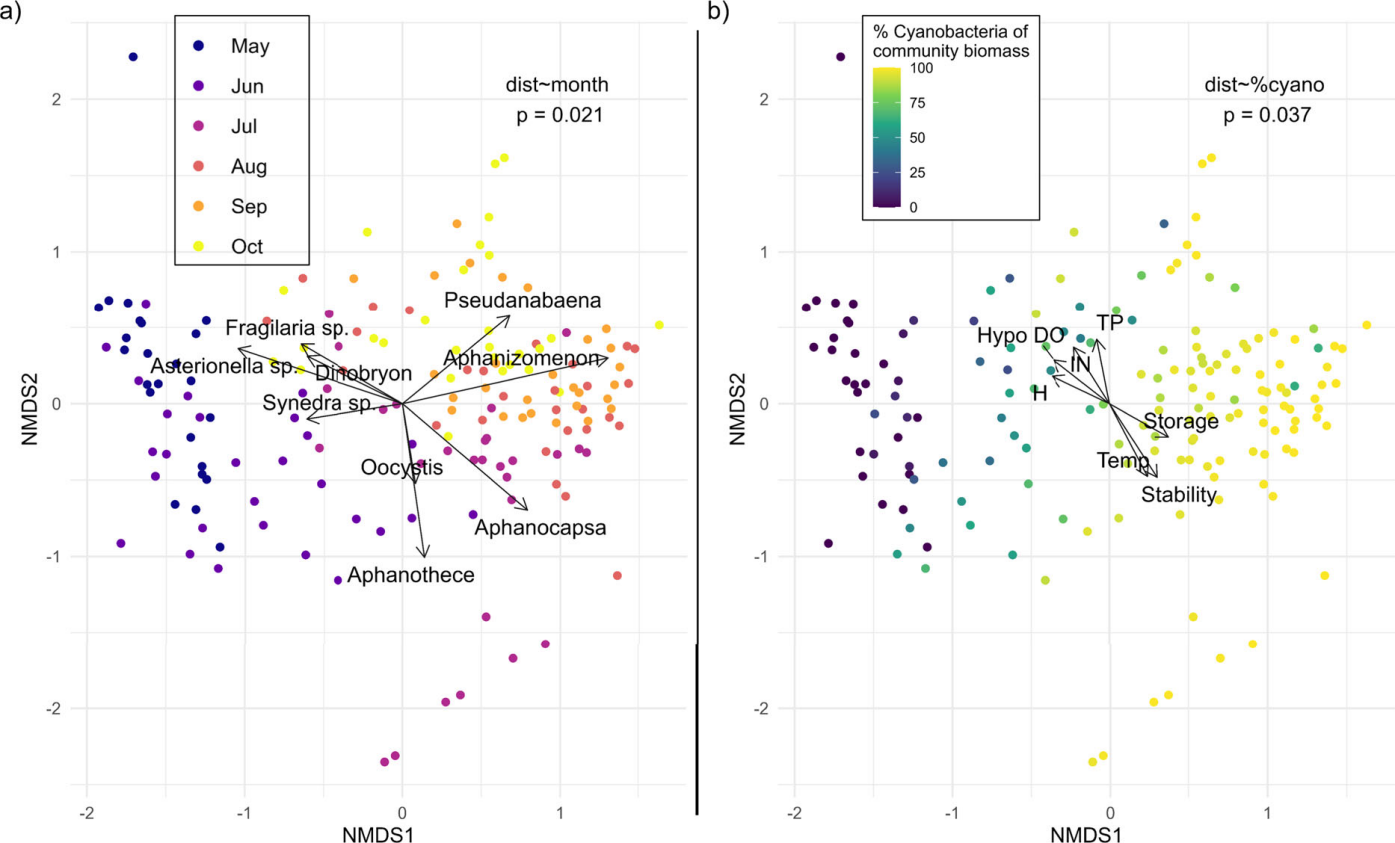
Beta diversity dispersion of phytoplankton communities in Boysen Reservoir, with each point representing a location and date. **a** Points colored by month, displaying the important control time has on diversity dynamics (PERMANOVA, dist month, *p*=0.021). Intrinsic variables overlayed with arrows representing the direction and magnitude of importance ($p<0.001$ for all phytoplankton visualized). **b** Points colored by the cyanobacteria percent of total community biomass, displaying the important control cyanobacterial varieties play in overall community diversity dynamics (PERMANOVA, dist %cyano, *p*=0.037). The extrinsic variables are overlayed with arrows representing the direction and magnitude of importance ($p<0.05$ for all variables). Arrow magnitudes on NMDS were multiplied by 2 for better visualization

Generally in Boysen Reservoir, cyanobacteria bio-mass dominates the phytoplankton community (Figure S6). Cyanobacteria density typically hits a peak mid-season (July–August), with distinctly high peaks occurring in the riverine zone of the reservoir (Fig. 8a). The shores and bays followed the riverine zone for high density. In 2021 and 2023, we observed high cyanobacterial density late season in the riverine zone, with peaks occurring in September and October. There were 77 phytoplankton taxa identified over the four years of monitoring. Of the 15 taxa with the highest relative abundance, eight were cyanobacteria (Fig. 8b), including the six most abundant taxa. *Aphanothece* topped the list, followed by *Aphanizomenon*. Over the 4 years of monitoring, 28 cyanotoxin producing blooms were confirmed, with the presence of anatoxin-a and/or total microcystins detected at each bloom (Figure S7). Overall, total microcystins were the most present toxins confirmed at blooms in Boysen Reservoir. In 2021, toxins were present July–October near Tough Creek Campground and Lacustrine Pelagic Dam. In 2022, toxins were detected in the riverine zone near Fremont Bay in July; then later during August–October, toxic blooms were present near Lacustrine Pelagic Dam, Tough Creek Campground, and Cottonwood Creek Bay. In 2023, toxin production was detected August-October near Lacustrine Pelgaic Dam and Cottonwood Creek Bay.

**Fig. 9:**
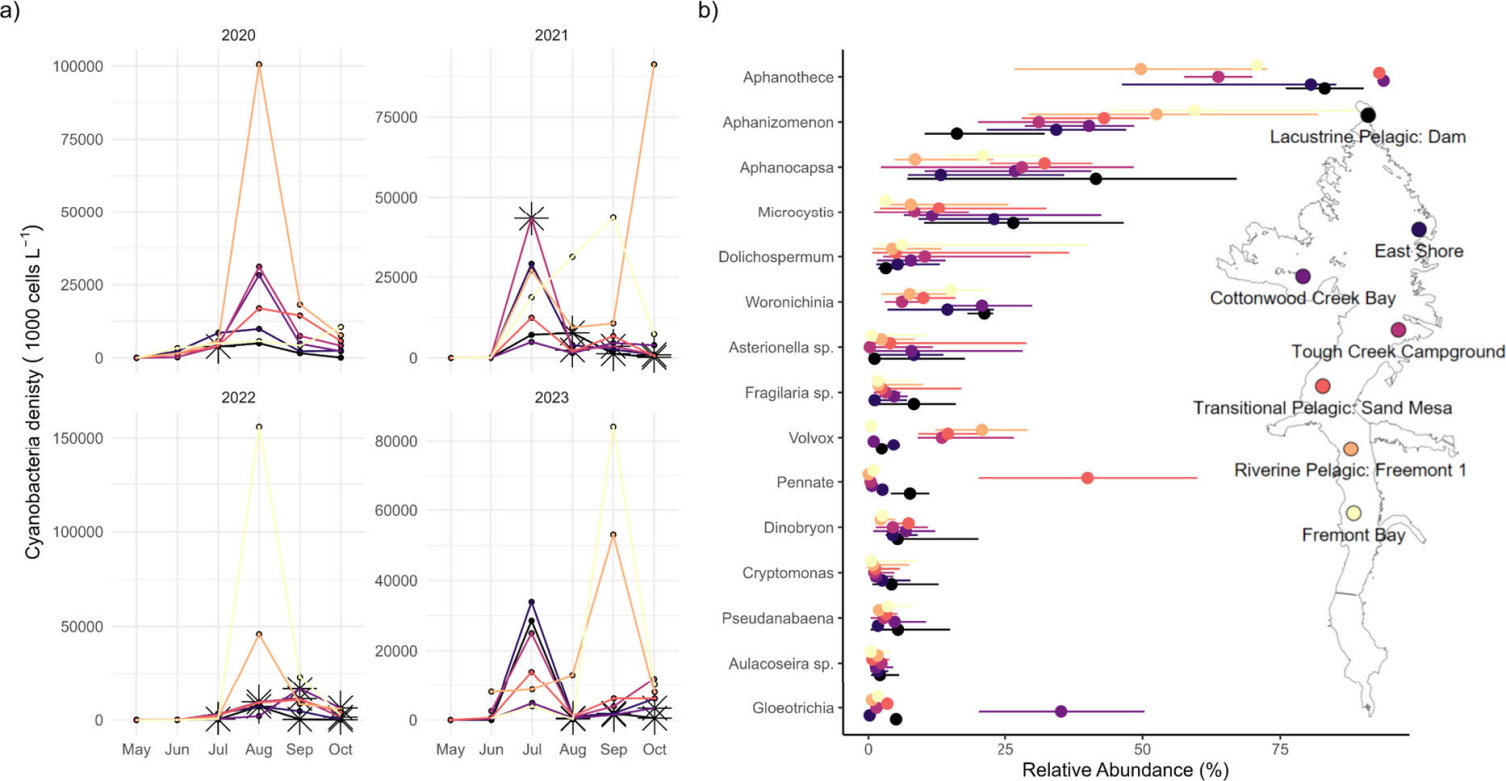
**a** Cyanobacteria density (represented in 1000 cells L-1) at each sampling date and location. Each panel shows one year, with the colors depicting location. Asterisks indicate cyanotoxin detection nearest that location. b Relative abundance of the 15 most abundant phytoplankton varieties at each sampling location. Abundance points are medians aggregated over time, with the error bars showing the 25th and 75th percentiles. Colors depict sampling locations shown in map

There were seven extrinsic variables that proved important to explain the community beta diversity (Fig. 7b). In one direction, toward earlier season and less cyanobacteria presence, we observed higher TP, IN, hypolimnion DO, and species diversity (*H*). In the opposite direction, monthly average reservoir storage, resistance to mixing (stability), and surface water temperature were all increased. Higher nutrient concentrations, especially IN, occurred in the same direction as higher species diversity and lower cyanobacteria, likely owing to the often N or co-limitation occurring in this reservoir (Figure S5). When cyanobacteria dominated mid-season, species diversity was low (Figure S8) and Schmidt stability was high (Figure S9).

## Discussion

In this study, we combined comprehensive monitoring efforts by the WY DEQ, USGS, and BoR to reveal how spatiotemporal patterns in reservoir water quality dynamics contribute to the proliferation of cyanobacteria and to uncover the key drivers of phytoplankton community shifts toward cyanobacterial dominance. Water quality dynamics were complex in Boysen Reservoir, yet we found strong temporal trends that accompanied the seasonal progression leading to cyanobacteria proliferation. Specifically, we found nutrient loading to be largely hydrologically driven, with snowmelt runoff transporting high nutrient inflows, especially P, and in-reservoir nutrient concentrations varied over time, likely with changes in hydrological and biological controls within the reservoir. Water quality dynamics differed across space, with the strongest differences between riverine and lacustrine zones and more homogenous patterns in the transitional zone, bays, and shores. However, changes in the phytoplankton community were not spatially driven. Rather, cyanobacteria consistently dominated Boysen Reservoir phytoplankton communities mid through late season and were strongly correlated with temporal drivers, like stratification and water temperature.

### Spatiotemporal trends in loading, nutrient limitation, and their influence on water quality

Robust monitoring programs have allowed for characterization of the spatiotemporally heterogeneous nutrient dynamics into, within, and exported from Boysen Reservoir. Nutrient loading was greatest in the primary Wind River Inlet, and concentrations within and exiting Boysen Reservoir were similar to that of this inlet (Figs. 2, 3, S2). Nutrient concentrations were highest in the two minor tributaries, but because nutrient loading was largely hydrologically driven with high streamflows during snowmelt runoff, these tributaries were less influential than the Wind River Inlet. High nutrient loading—both chronically and episodically high—has been linked to HABs globally, and nutrient loading reductions have proven successful in decreasing HAB production (Heisler et al., 2008). In some cases, monitoring the major water quantity tributaries may be a good strategy for determining overall nutrient inputs, rather than monitoring tributaries with the highest concentrations. This, however, can be complicated as the number of small tributaries increases, so too does their combined influence (Mooney et al., 2020). Nutrient outflow of Boysen reflected a dampening effect of the reservoir, in which concentrations and stoichiometry were similar to that of the reservoir. Loading heterogeneity was especially realized via nutrient stoichiometry (Fig. 4), in which tributary TN:TP ratios were depleted April–September compared to October–March.

The striking stoichiometric pattern in the tributaries follows tributary TP concentrations, which are highest April–September (Figure S3), likely owing to hydrologic increases importing more P relative to N, shifting the tributaries to N-limitation during the growing season. This pattern agrees with the general pattern of seasonal hydrological drivers of nutrient stoichiometry seen in other watersheds, with peak N:P in winter and low N:P spring-fall due to enhanced P transport (Green & Finlay, 2010). Controls on nutrient stoichiometry vary between tributaries and lakes leading to significant differences between them. Tributaries are more likely to be influenced by water availability and terrestrial inputs while lakes have more complex controls like mixing, sedimentation, internal cycling, and temperature (Prater et al., 2017; Rogora et al., 2021). In semi-arid watersheds, similar to that of Boysen Reservoir, there may be decoupling of terrestrial-aquatic biogeochemical linkages, with snowmelt runoff as the main driver of seasonal N:P variation (Green & Finlay, 2010), consistent with our observations of low tributary N:P during the spring and summer when discharge is greatest.

Nutrient concentrations within Boysen Reservoir demonstrated varying temporal patterns, as N (both TN and inorganic N) increased as the growing season progressed, while P (both TP and phosphate) showed more dynamic pattern with minimal concentrations mid-season (Fig. 3). These patterns were matched by internal nutrient retention (Figure S4), in which N was increasingly retained in the reservoir as the season progressed, potentially due to cyanobacterial N-fixation and internal nutrient cycling (Xu et al., 2021). P retention tended to be highest near the timing of concentration minima, a sign of biotic use or sedimentation. Reservoirs fundamentally change the movement of water, nutrients, and aquatic biota as dams alter hydrology, biology, and chemistry of a system (Hayes et al., 2017). Dammed reservoirs can serve as a P sink because of increased sedimentation (Wu et al., 2016), and sedimentation may be a driver of better water quality within reservoirs compared to their tributaries (de Oliveira et al., 2021). Yet on an annual scale (with the exception of 2020, in which we observed the lowest streamflows during the four years of monitoring), we found generally greater P export from the reservoir and greater N-retention. This is likely a combination of hydrological and biological controls on nutrient cycling and the overall modulating effect of the reservoir as the outflow conditions match those of the reservoir.

Despite higher N-retention, primary productivity in Boysen Reservoir was typically N-limited or co-limited by both N and P or other confounding factors (Figure S5). N-limited conditions can lead to higher abundances of N-fixing species that produce cyanotoxins (Smith, 1983; Gobler et al., 2016; Paerl & Otten, 2016; Paerl & Scott, 2010), thus altering overall water quality and increased N concentrations. Globally, assembly shifts toward cyanobacteria in eutrophic lakes are driven by both N-fixation and temperature, but once hypereutrophication is reached, temperature becomes the most important factor (Tanvir et al., 2021). Water quality monitoring is increasingly important as climate change is predicted to influence nutrient and stoichiometry dynamics as warming air temperatures will intensify nutrient enrichment and alter retention and export (Farrell et al., 2020). Monitoring both N and P for eutrophication management provides crucial information about potential for primary productivity, especially as N:P can vary over space and time and often lakes are co-limited (Rock & Collins, 2024). Combined reductions of N and P are likely the most productive management strategy for controlling eutrophication.

Large reservoirs can show distinct spatial variability in water quality across the three zones. For example, a large tropical Brazilian reservoir demonstrated how precipitation heavily influenced water quality in the riverine zone while the lacustrine zone was most influenced by stratification (Nogueira et al., 1999). Water quality in Boysen Reservoir was spatiotemporally heterogenous with patterns that persisted year to year (Fig. 5). Particularly, we observed distinct differences between the lacustrine and riverine zones, but there may be less variability between the intermediate zone sites and the shore/bay sites than when comparing the three zones overall. Previous studies show inconsistent patterns of surface water quality heterogeneity in reservoirs, from lateral homogeneity (Wells & Gordon, 1982) to concentration gradients along the reservoir (Lindim et al., 2011). However, this may be in part due to reservoir shape. In the case of the gradient, the reservoir showed homogeneity after a constriction in which the reservoir’s shape became less riverine and more like a basin (Lindim et al., 2011). Thus, it is important to consider the complex shape of reservoirs when determining where to monitor water quality parameters.

Temporal patterns in Boysen Reservoir consistently show physicochemical and nutrient patterns that complement cyanobacterial presence. Notably, the riverine and lacustrine zones displayed the most diverging patterns. This distinction is especially prevalent in PC3, with the lacustrine zone showing greater Secchi depth, stability, maximum depth, and lower hypolimnion temperature than the rest of the reservoir. Fremont Bay consistently displayed opposite the Lacustrine Pelagic Dam on PC1 and PC3, concurrent with more intense cyanobacteria presence in the riverine zone (Fig. 8). In the lacustrine zone during July–October, when cyanobacterial blooms form, we observed an increase in phosphate potentially from internal loading as Schmidt stability and surface temperature decreased (Lacustrine Pelagic Dam migrates right in PC1 space). Cyanobacteria have shown dominance in waters with low N:P ratios (Smith, 1983), like we observed in Boysen Reservoir. In October, when resistance to mixing was low (Figure S9), the sites were becoming more similar with the exception of the lacustrine zone, which maintained higher stability, Secchi depth, and lower hypolimnion temperature compared to the rest of the reservoir, indicating this site will be the last to mix with the rest of the reservoir. The consistency of water quality patterns year to year provides confidence behind these general spatiotemporal patterns in Boysen Reservoir. Combined with phytoplankton community data, we were able to better understand the drivers of community shifts toward cyanobacteria.

### Challenges in predicting primary productivity drivers in a cyanobacteria-dominated reservoir

Monthly monitoring for phytoplankton identified to genus or species taxonomic level, when possible, provided substantial information about when, where, and who was present and dominant in the communities. The phytoplankton community in Boysen Reservoir was dominated by cyanobacteria (Figs. 8, S6), which typically were at peak biomass mid-season (July–August), especially in the riverine zone. In opposition to our hypothesis that reservoir zones would in part drive community dynamics, there was no significant spatial variability (Fig. 6). Zooplankton communities have demonstrated distinct heterogeneity associated with reservoir zones (Bini et al., 1997). Rather, the domination by cyanobacteria was closely related to the passing of the season (Fig. 7). Cyanobacteria dominance in freshwaters worldwide is a predicted consequence of climate change due to their unique physiological features, including affinity for high temperatures, buoyancy, ability to store P for later use, ability to fix N, dormancy over winter, and low-light adaptability (Carey et al., 2012). Although identification to the lowest taxonomic level was not necessary for observing the abrupt shift in community diversity to cyanobacterial dominance, it is critical for future studies investigating toxin production by various cyanobacterial species.

In May and June, Boysen Reservoir was dominated by diatoms and golden algae and had higher species diversity (*H*) overall. Higher nutrients, especially IN, occurred in the same direction as higher species diversity. The greater biologically available N was likely important to support a diverse community in a reservoir that was often N-limited (Figure S5). The community quickly transitioned toward cyanobacteria in July, with high prominence of N-fixing *Aphanothece* and *Aphanizemenon*. Notably, this trend was consistent across all four years of monitoring. This level of stable community composition and temporal variability has been observed in other reservoirs; with the strongest drivers influencing communities being water retention (Soares et al., 2012), stratification, and water level (Yang et al., 2018). We found the strongest drivers of the community shift to cyanobacteria dominance were reservoir storage, stability, and temperature. Similar drivers of cyanobacteria presence were found in a previous study in a Chinese reservoir with summertime cyanobacteria dominance. They additionally found phytoplankton communities were more variable vertically than horizontally across the surface (Lv et al., 2013).

Despite the high spatial and taxonomic resolution of phytoplankton community sampling in Boysen Reservoir, vertical phytoplankton community data may help us to even better understand dynamics below the surface that set up for HABs. Coupling between pelagic and benthic communities aids the proliferation of the cyanobacterial species year after year (Verspagen et al., 2005). Certain cyanobacterial taxa have resting cell stages or akinetes, which can lead to recurring blooms annually. In such stages, cyanobacteria can overwinter and re-inoculate the following year (Carey et al., 2012). For example, simulation of a Microcystis population showed pelagic blooms could be reduced by 50% if resting cells in the benthos could be removed, and by > 60% if the pelagic resting cells could be flushed (Verspagen et al., 2005). Reservoir storage was one of the primary factors driving high percentage of cyanobacteria in the phytoplankton community in Boysen Reservoir (Fig. 7b), suggesting the higher, more stable water level may allow resting cell stages to remain viable. Sampling vertically for phytoplankton can address questions around pelagic-benthic coupling of the community.

Primary productivity, especially over-productivity that can result in toxin production, is attributed to com-plex interactions between nutrient dynamics, light avai-lability, and temperature. Other factors like predation and competition are also important for understanding productivity and phytoplankton community dynamics (Likens, 2010). Toxin production has been shown to be complex spatially in waters with complex physical structures (Smith et al., 2020) as well as within communities where multiple cyanobacteria are present and competing (Christensen et al., 2022). In a community so overtly dominated by cyanobacteria, underscoring drivers of community shifts can be especially difficult as the diversity is low. In our study years, toxin detection did not always correlate with the highest density of cyanobacteria (Fig. 8a). *Aphanizomenon*, *Dolichospermum*,*Woronichinia*, *Aphanocapsa*, and *Mi-crocystis*—among the most abundant phytoplankton identified in our study years (Fig. 8b)—are known to produce several neurotoxins, including anatoxin-a and/or microcystins (Carmichael & Boyer, 2016). *Aphanothece*, the most abundant variety in our study, have also been associated with microcystins production (Dasey et al., 2005). Cyanotoxin sampling in Boysen Reservoir occurred along the shoreline, mainly at beaches and boat ramps, when a HAB was suspected. Testing for toxins at each of the monitored locations may provide better insight into HAB production and the warning signs of toxic compounds.

Nutrient loading to Boysen Reservoir was high across all years, exacerbating the high biomass of cyanobacteria and cyanotoxin production. TP concentration range of the tributaries to Boysen were similar to those seen in midwestern tributaries to Lake Michigan, yet the TN concentration range was more constrained compared to those same streams influxing to Lake Michigan (Mooney et al., 2020). Reduction of both N and P to control HABs is likely the most productive nutrient management strategy (Tanvir et al., 2021; Paerl et al., 2018). Under-ice water quality dynamics in northern lakes can also play a large role in nutrient concentrations and primary productivity both during winter and into the summer (Wen et al., 2020; Kalinowska et al., 2019), and ice and snow quality can alter under-ice oxygen availability (Gorsky et al., 2024) and phytoplankton community structure (Socha et al., 2023). Increased monitoring of this eutrophic reservoir to year-round would likely illuminate how seasonal variability contributes to the ongoing HABs.

## Conclusion

Through extensive monitoring conducted by three state and federal institutions, the data presented here underscore the importance of spatiotemporal monitoring to understand complex dynamics of nutrient loading and phytoplankton communities in reservoirs. We identified trends in nutrient inputs, retention, and phytoplankton shifts, revealing links between water quality drivers and the conditions that foster HABs in Boysen Reservoir. Comprehensive monthly monitoring of multiple locations within and surrounding the reservoir provided capacity to identify key drivers of cyanobacterial abundance. Such detailed data can help us move beyond HAB detection toward proactive management, guiding adaptive monitoring programs that can allocate resources to mitigate the primary sources of loading and consider the physicochemical conditions under which HABs are occurring. Through these efforts, we hope to equip managers with strategies to monitor and safeguard water resources amid escalating climate pressures and ultimately to reduce HAB frequency and impact.

## Supplementary Information

Below is the link to the electronic supplementary material.

Supplementary file 1 (pdf 6657 KB)

## Data Availability

Data and code to perform analyses and create manuscript figures are located at https://zenodo.org/records/15685445.

## Declarations

### Disclaimer

Any use of trade, firm, or product names is for descriptive purposes only and does not imply endorsement by the US Government.
