# Supplementary material for: Spatiotemporal drivers of water quality and phytoplankton communities in a cyanobacteria-dominated reservoir provide management insights
Source: Environ Monit Assess. 2025 Jun 24;197(7):795. doi: 10.1007/s10661-025-14258-1 (PMC12185601; doi:10.1007/s10661-025-14258-1)
Supplement: Supplementary file 1 — (pdf 6657 KB) [file 10661_2025_14258_MOESM1_ESM.pdf]

Supporting Information for: Spatiotemporal drivers of water quality and phytoplankton communities in a cyanobacteria-dominated reservoir provide management insights

Linnea A. Rock[1 2], William W. Fetzer[1 2], Lindsay S. Patterson[3], Samuel J. Sillen[4], Ron Steg[3], Annika W. Walters[1 2 5], and Sarah M. Collins[1 2]

[1]Department of Zoology and Physiology, University of Wyoming, Laramie, WY, USA

[2]Program in Ecology and Evolution, University of Wyoming, Laramie, WY,USA

[3]Water Quality Division, Wyoming Department of Environmental Quality, Cheyenne, WY, USA

[4]Department of Geology and Environmental Science, University of Pittsburgh, Pittsburgh, PA, USA

[5]USGS Wyoming Cooperative Fish and Wildlife Research Unit, University of Wyoming, Laramie, WY, USA

Corresponding author: L.A. Rock, lrock@uwalumni.com

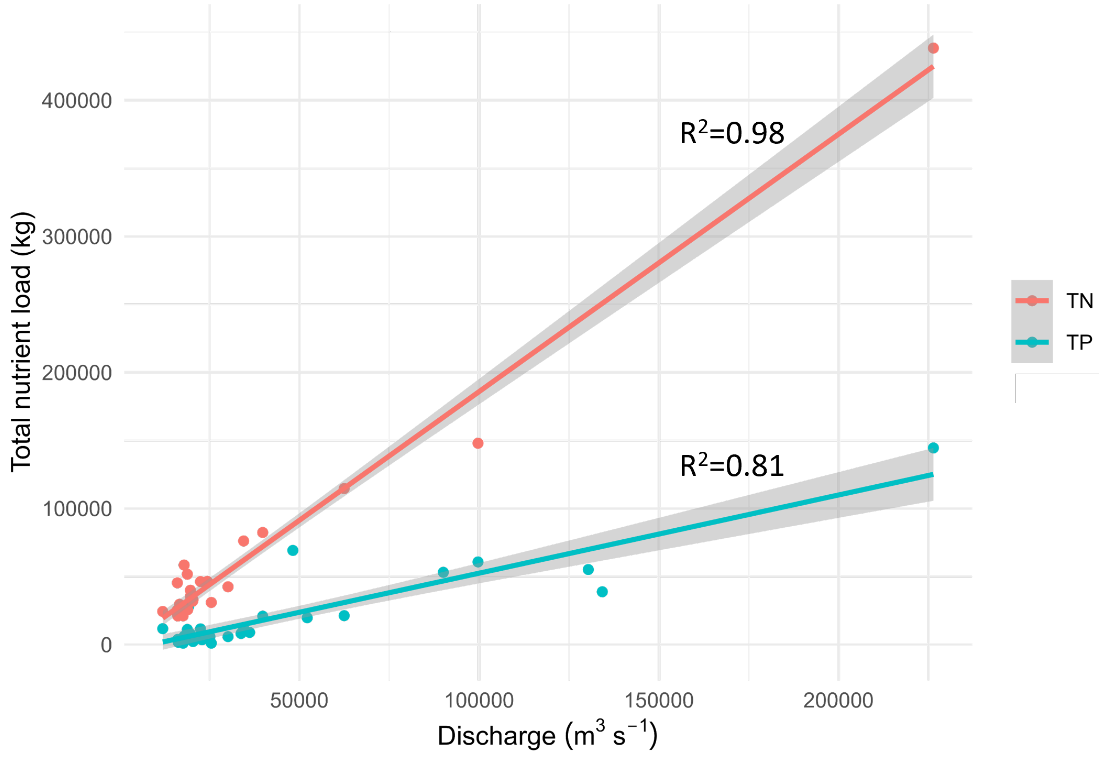

Figure S1: Linear models correlating the sum of total nitrogen (TN, red) and sum of total phosphorus (TP, blue) monthly tributary loading to Boysen Reservoir with the sum of tributary discharge. Corresponding R2 displayed next to each line.

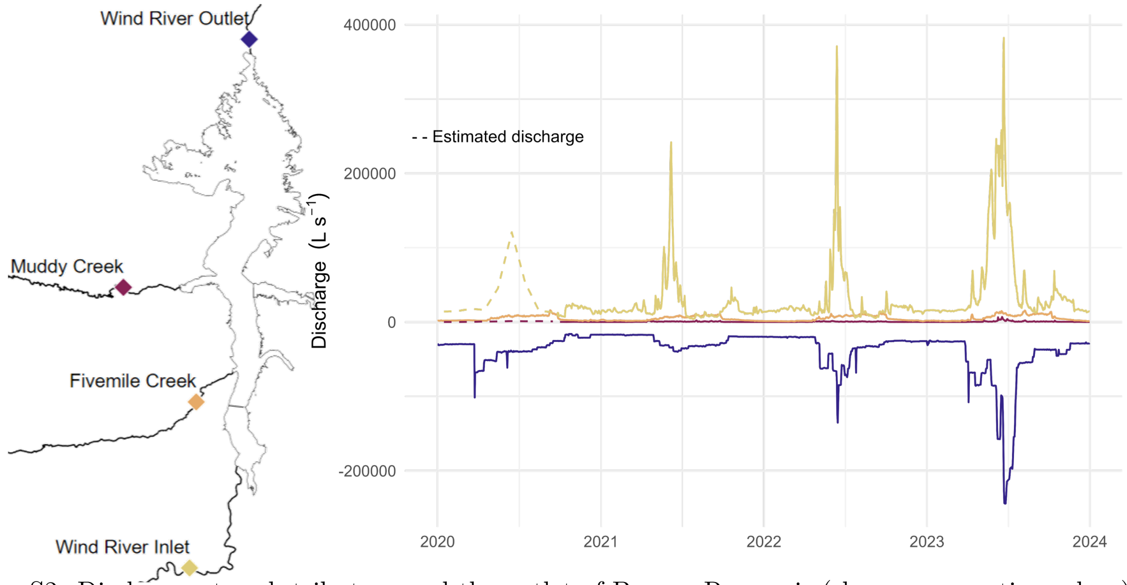

Figure S2: Discharge at each tributary and the outlet of Boysen Reservoir (shown as negative values) from January 2020 - December 2023. Dotted lines January-September 2020 for Wind River Inlet and Muddy Creek were interpolated as the historical monthly averages. Legend shows the location of each tributary and the outlet.

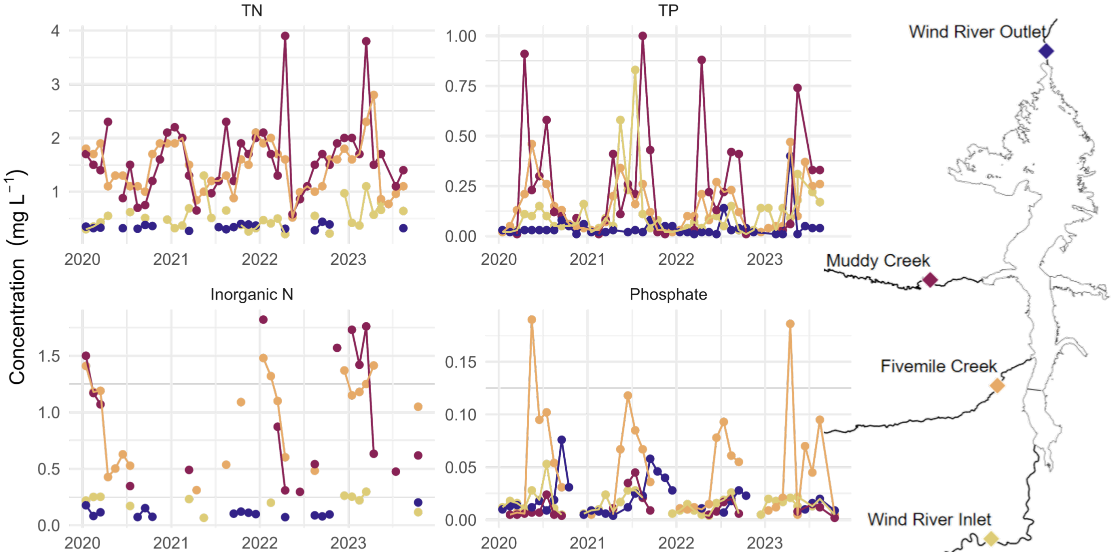

Figure S3: Total nitrogen (TN), total phosphorus (TP), inorganic nitrogen (N), and phosphate monthly nutrient concentrations in each tributary and the outlet. Legend shows the location of each tributary and the outlet.

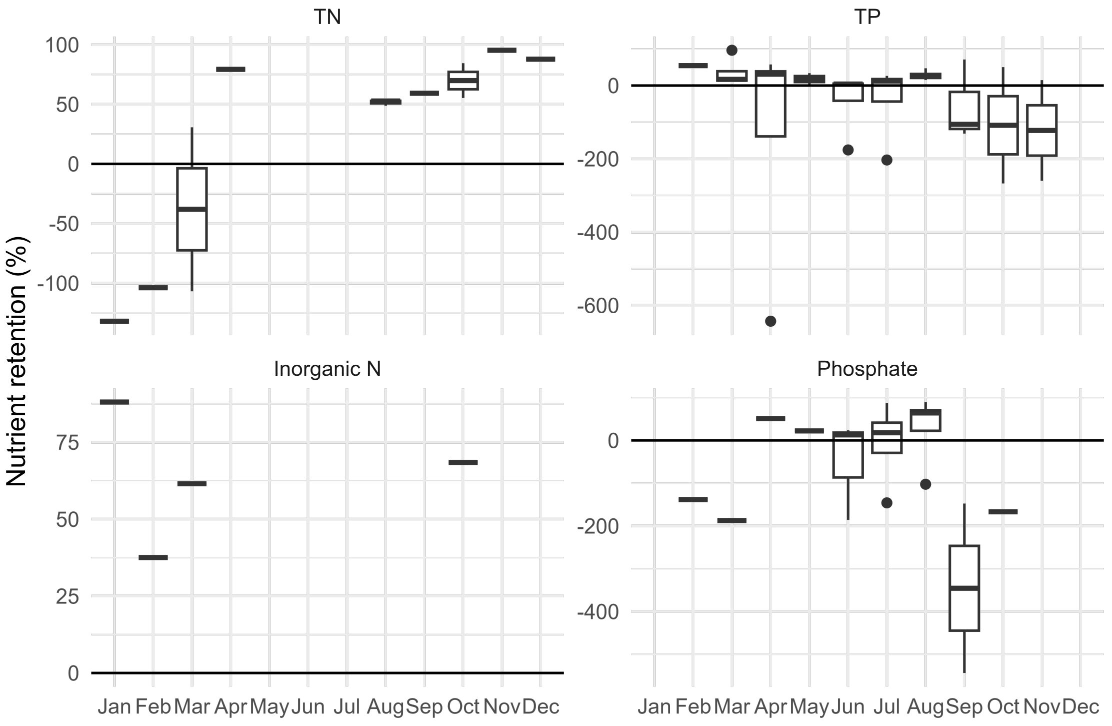

Figure S4: Monthly nutrient retention over the year for Total nitrogen (TN), total phosphorus (TP), inorganic nitrogen (N), and phosphate.

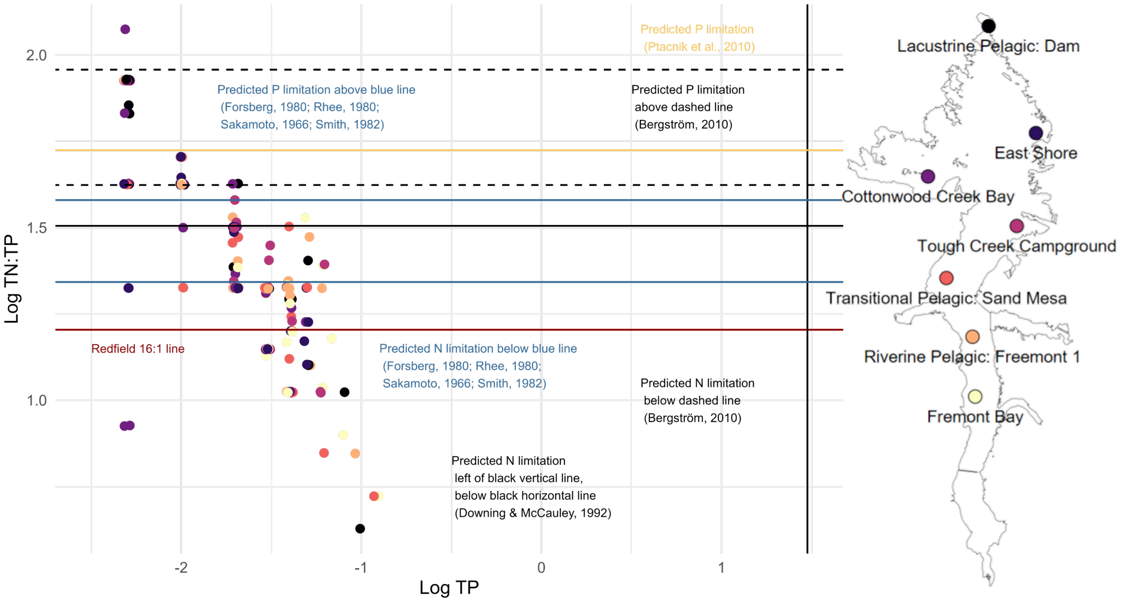

Figure S5: Nutrient in Boysen Reservoir based on the ratio of total N: total P. We compared N:P ratios limitation at each sampling location and date to 8 different published N:P ratios, which are described on the figure. The sampling site legend is plotted over the outline of Boysen Reservoir for context on the position of each site.

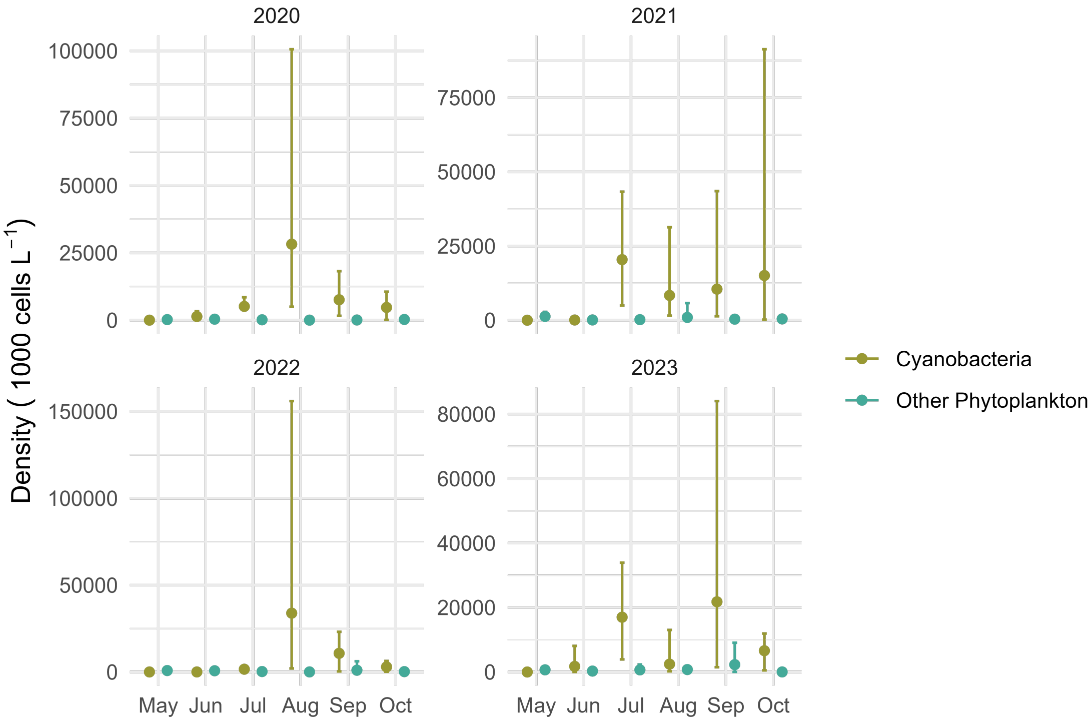

Figure S6: Cyanobacteria density (as 1000 cells L-1) compared to the density of other phytoplankton monthly. Each panel represents a different sampling year. Points are monthly mean aggregated across the sampling sites and bars show the minimum and maximum values observed that month.

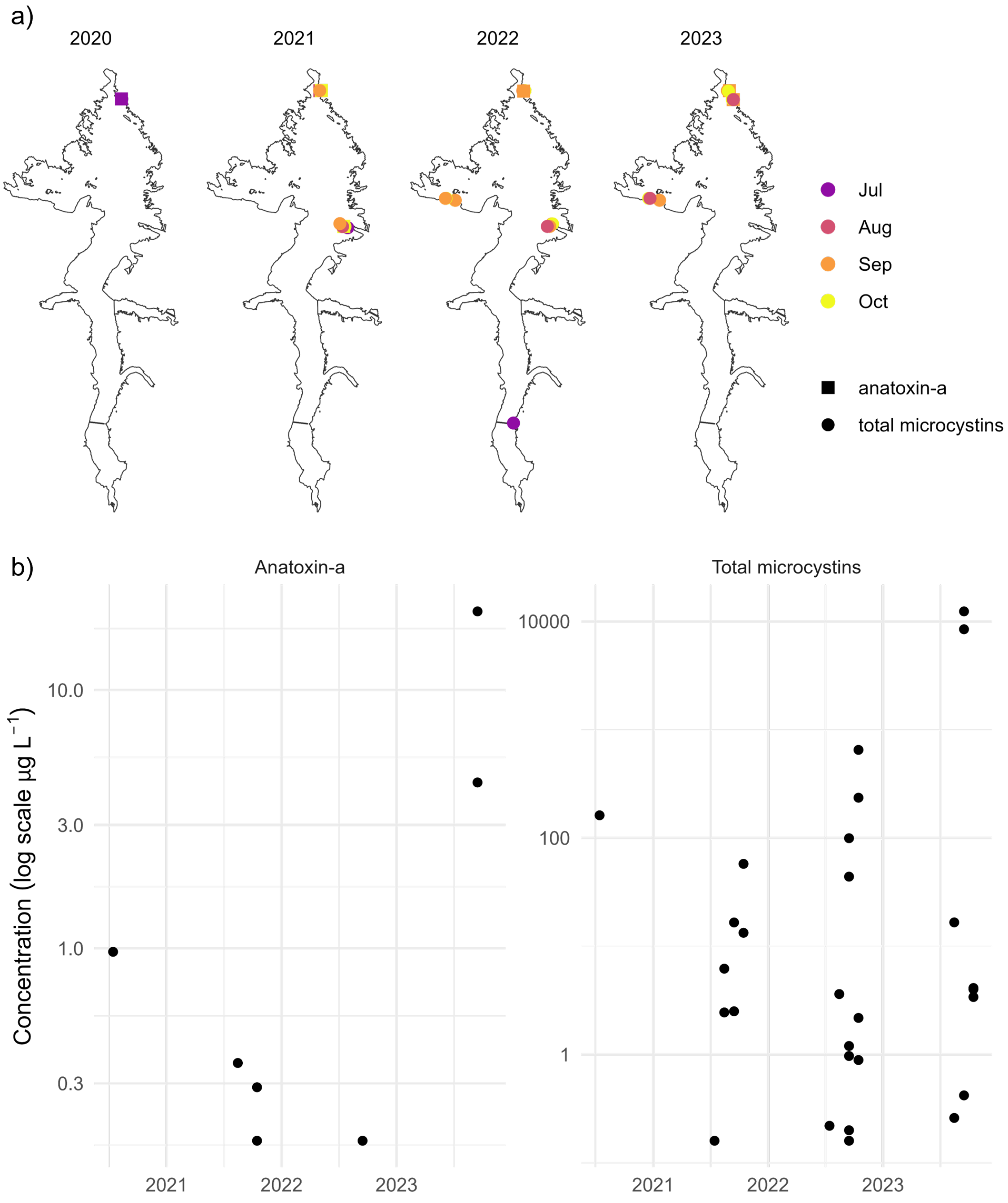

Figure S7: Cyanotoxin presence in Boysen Reservoir. a) Locations of cyanotoxin detection colored by the month of detection. Squares denote presence of anatoxin-a, while circles denote presence of total microcystins. b) Concentration, shown on the log scale, of anatoxin-a in (first panel) and total microcystins (second panel) at each detection.

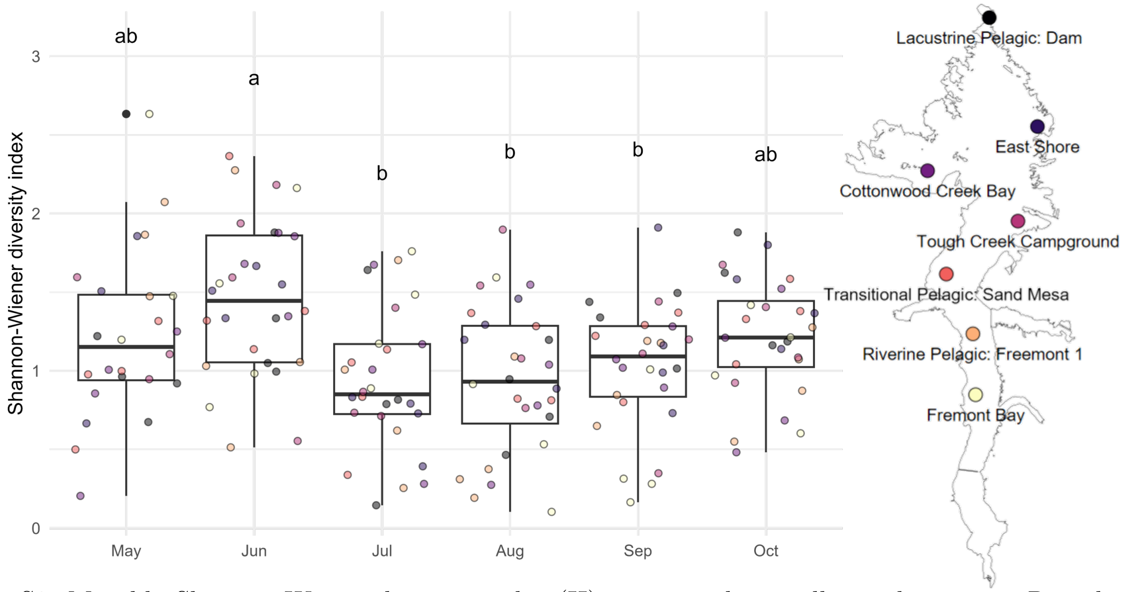

Figure S8: Monthly Shannon-Wiener diversity index (H) aggregated over all sampling years. Raw data are depicted as points with each sampling location represented by a different color. Lettering above boxplots denotes significance of difference in medians (horizontal bars on boxplots) between months. The sampling site legend is plotted over the outline of Boysen Reservoir for context on the position of each site.

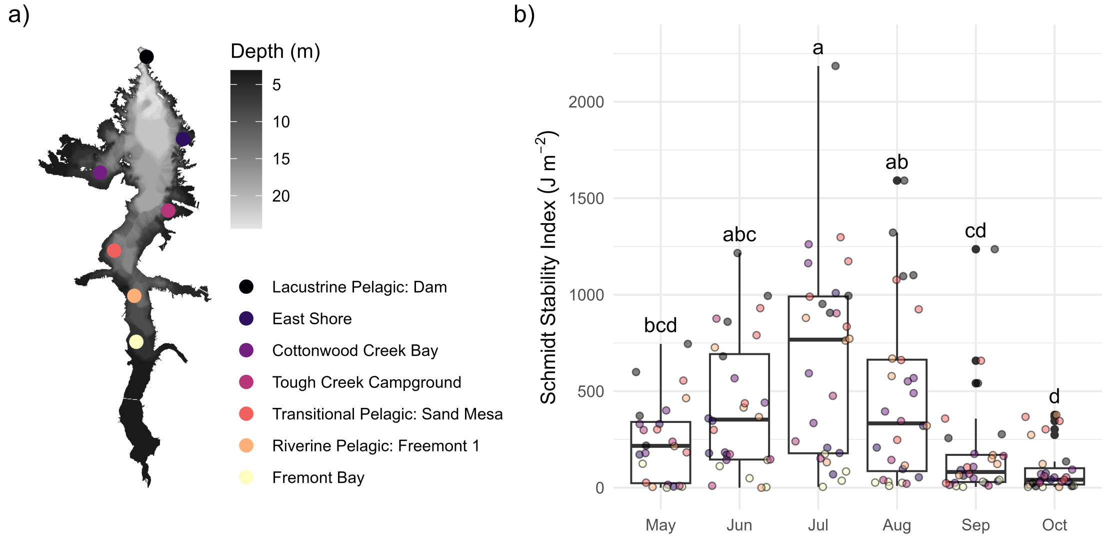

Figure S9: Bathymetry plot of Boysen Reservoir to display the varying depth context at the 7 sampling locations. b) Monthly Schmidt stability, or the force required to mix the column of water aggregated over all sampling years. Raw data are depicted as points with each sampling location represented by a different color. Lettering above boxplots denotes significance of difference in medians (horizontal bars on boxplots) between months.
